# Snap-through transition of buckled graphene membranes for memcapacitor applications

**DOI:** 10.1038/s41598-018-21205-3

**Published:** 2018-02-23

**Authors:** Ruslan D. Yamaletdinov, Oleg V. Ivakhnenko, Olga V. Sedelnikova, Sergey N. Shevchenko, Yuriy V. Pershin

**Affiliations:** 1 Nikolaev Institute of Inorganic Chemistry SB RAS, Novosibirsk, 630090 Russia; 2 Novosibirsk State University, Novosibirsk, 630090 Russia; 3 B. I. Verkin Institute for Low Temperature Physics and Engineering, Kharkov, 61103 Ukraine; 4 V. N. Karazin Kharkov National University, Kharkov, 61022 Ukraine; 5 Department of Physics and Astronomy, University of South Carolina, Columbia, South Carolina 29208 USA

## Abstract

Using computational and theoretical approaches, we investigate the snap-through transition of buckled graphene membranes. Our main interest is related to the possibility of using the buckled membrane as a plate of capacitor with memory (memcapacitor). For this purpose, we performed molecular-dynamics (MD) simulations and elasticity theory calculations of the up-to-down and down-to-up snap-through transitions for membranes of several sizes. We have obtained expressions for the threshold switching forces for both up-to-down and down-to-up transitions. Moreover, the up-to-down threshold switching force was calculated using the density functional theory (DFT). Our DFT results are in general agreement with MD and analytical theory findings. Our systematic approach can be used for the description of other structures, including nanomechanical and biological ones, experiencing the snap-through transition.

## Introduction

Memcapacitors^1^ are an emerging type of circuit elements with memory whose instantaneous response depends on the internal state and input signal. Such devices are prospective candidates for applications in information storage and processing^2,3^ technologies as their states can be manipulated by the applied voltages or charges and can store information for long intervals of time. Several possible realizations of memcapacitors were suggested by using micro-electro-mechanical systems^4^, ionic transport^5^, electronic effects^6^, superconducting qubits^7^, etc^8^.

Generally, voltage-controlled memcapacitive systems (memcapacitors) are described by^1^

1 $$q(t)=C(x,V,t)V(t),$$

2 $$\dot{x}=f(x,V,t),$$

where *q*(*t*) is the charge on the capacitor at time *t*, *V*(*t*) is the applied voltage, *C* is the *memcapacitance* (memory capacitance), *x* is a set of *n* internal state variables, and *f* is a continuous *n*-dimensional vector function. In some cases, it is more convenient to consider charge-controlled memcapacitors^1^ such that the charge instead of voltage is considered as input.

**Figure 1:**
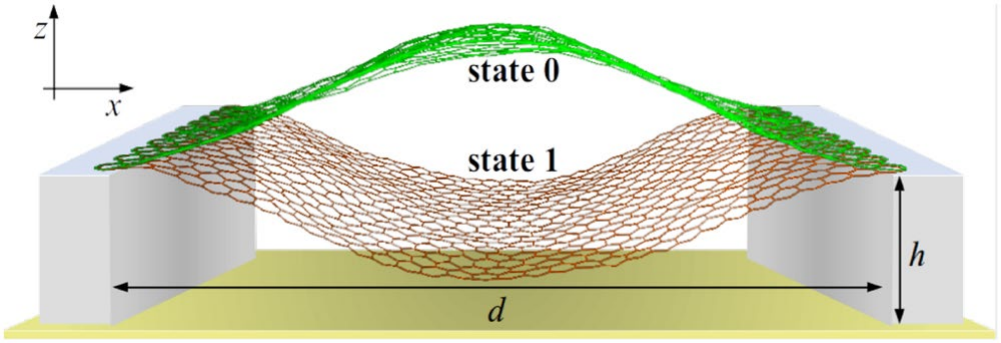
Schematics of the membrane memcapacitor employing a buckled graphene membrane as its top plate^4^.

Among several possible realizations of memcapacitors, the membrane-based memcapacitors^4^ are of significant interest as their geometry makes them intrinsically suitable for non-volatile storage of binary information. Indeed, the buckled membrane used as the top capacitor plate (see Fig. 1 for schematics) has two stable buckled states corresponding to two distinct values of capacitance. It was suggested^4^ that the switching between these states can be performed using the attractive interaction of oppositely charged capacitor plates. Moreover, it was demonstrated theoretically that simple circuits of membrane memcapacitors offer an in-memory computing functionality^3^.

In this work, we consider a possible realization of membrane-based memcapacitor^4^ employing a single- or multi-layer graphene membrane^9–13^ as its bistable plate (see Fig. 1). Our aim is to understand the basic physical processes and parameters underlying the snap-through transition of such membrane including details of the membrane dynamics, threshold forces, etc. For this purpose, we perform a combined study using MD, DFT and elasticity theory focusing on a single-layer graphene membrane with clamped boundary conditions. This choice of boundary conditions is justified by the typically strong adhesion of graphene to substrates. Our results extend our prior DFT investigation^14^ of the up-to-down snap-through transition of graphene membrane with hinged boundary conditions.

The combination of computational/theoretical methods adds breadth and depth to our analysis. Using MD simulations, we were able to understand main features of the membrane dynamics in the presence of an external force and after the force removal. This understanding has helped us to develop analytical models that resulted in compact algebraic expressions for the threshold switching forces. DFT calculations were used to validate MD results for the up-to-down transition.

This paper is organized as follows. In Sec. “Molecular Dynamics Simulations” we investigate the snap-through transition of graphene membranes using molecular dynamics simulations. In particular, MD simulations of the up-to-down and down-to-up transitions are reported in Subsec. “Up-to-down transition” and “Down-to-up transition”, respectively, while MD simulation details can be found in Supplementary Information (SI) Sec. “MD Simulation Details”. The standard elasticity theory is applied to the membrane switching in Subsec. “Buckling and snapping-through within the theory of elasticity”. A phenomenological analytical model of the snap-through transition is presented in Subsec. “Phenomenological elasticity theory” and in SI Sec. “Phenomenological Elasticity Theory”. Our DFT calculations are summarized in Sec. “Density Functional Theory”. The results obtained within different approaches as well as their implications are discussed in Sec. “Discussion”.

In this paper, the following notations are used:

*q* - the charge on capacitor (see Eq. (1))

*V* - the applied voltage (see Eqs (1) and (2))

*C* - the (memory) capacitance (see Eq. (1))

*d* - the distance between fixed sides of membrane (see Fig. 1)

*h* - the distance between the bottom plate and the level of fixed sides (see Fig. 1)

*L* - the membrane length

*w* - the membrane width

*D* = 16 eV - the bending rigidity of graphene

*E*_2*D*_ = 340 N/m - the 2D Young’s module

*ζ* - the deflection of membrane (see Eq. (3))

*ζ*_c_ - the maximum deflection of membrane (see Eq. (7))

*θ*_*i*_(*s*) - the angle that the membrane makes with the horizontal (see Eqs (16) and (17)),

*s* - the internal coordinate that changes between −1/2 and 1/2 (see Eqs (16) and (17))

*A*_*i*_ and *c*_*i*_ - coefficients (see Eqs (16) and (17))

*z*_*cm*_ - the center of mass position (see Eq. (19))

*U*_*b*_, *U*_*str*_, *U*_*ext*_ - the bending, stretching and external potential contributions to the potential energy of membrane (see Eq. (18))

*F*^↓^ - the up-to-down threshold switching force (see Eqs (12), (20), (21) and (22))

*F*^↑^ - the down-to-up threshold switching force (see Eqs (14) and (28))

*ε*_0_ - the vacuum permittivity

## Molecular Dynamics Simulations

MD simulations are a well established modeling tool frequently employed in studies of nanoscale carbon-based materials^15–36^. We used classical MD simulations to investigate the dynamics of snap-through transition of buckled graphene membranes. Zigzag graphene nanoribbons (membranes) of two lengths were considered: the nanoribbon ***A***, *L* = 54 Å (22 rings), and nanoribbon ***B***, *L* = 103 Å (42 rings). Both nanoribbons were of the same width (*w* = 41 Å). In order to implement the clamped boundary conditions, the first two lines of carbon atoms at shorter sides were kept fixed. The buckling was realized by changing the distance between the fixed sides from *L* to *d* < *L*. The same external force was applied to each atom in the downward direction to simulate the attractive interaction between the plates.

See SI Sec. “MD Simulation Details” for the details of MD simulations.

### Up-to-down transition

**Figure 2:**
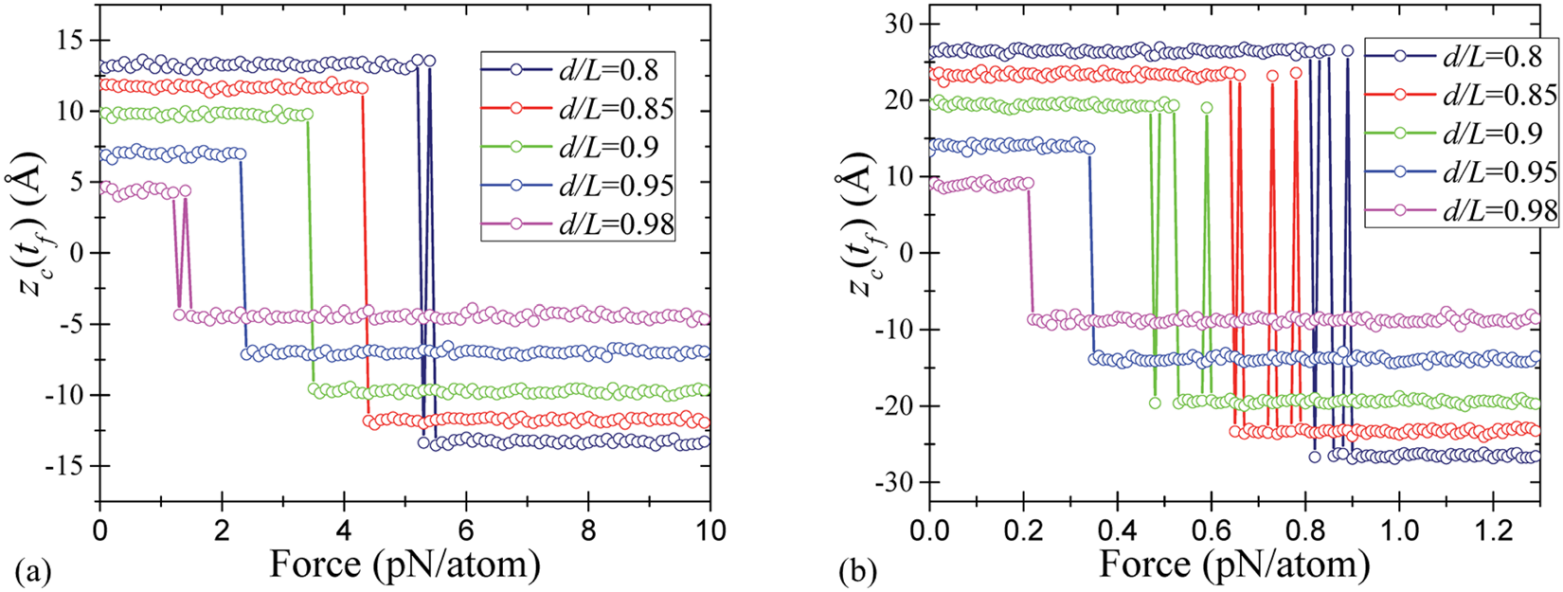
Up-to-down transition: the final position (at a time *t*_*f*_) of a central atom of membrane as a function of the applied force magnitude for (**a**) shorter membrane *A* (22 rings length), and (**b**) longer membrane *B* (42 rings length). The calculation details are given in the text.

In order to simulate the up-to-down transition, the double-clamped graphene nanoribbon buckled upwards was subjected to the force in the downward direction. The final state of membrane was found using MD simulations as described in SI Sec. “MD Simulation Details”. Figure 2 shows the final position of a central atom of membrane versus the applied force for several values of *d*/*L* and two membrane sizes. According to Fig. 2, at fixed *L*, the up-to-down threshold switching force (the minimal force required for the up-to-down transition) is larger for smaller values of *d*/*L*. Moreover, at fixed *d*/*L*, the threshold switching force is smaller for longer membranes. Additionally, some curves in Fig. 2 exhibit a noisy threshold (such as *d*/*L* = 0.8 and *d*/*L* = 0.98 in (a)), which can be related to thermal fluctuations of membranes. All these observations are intuitively reasonable.

**Figure 3:**
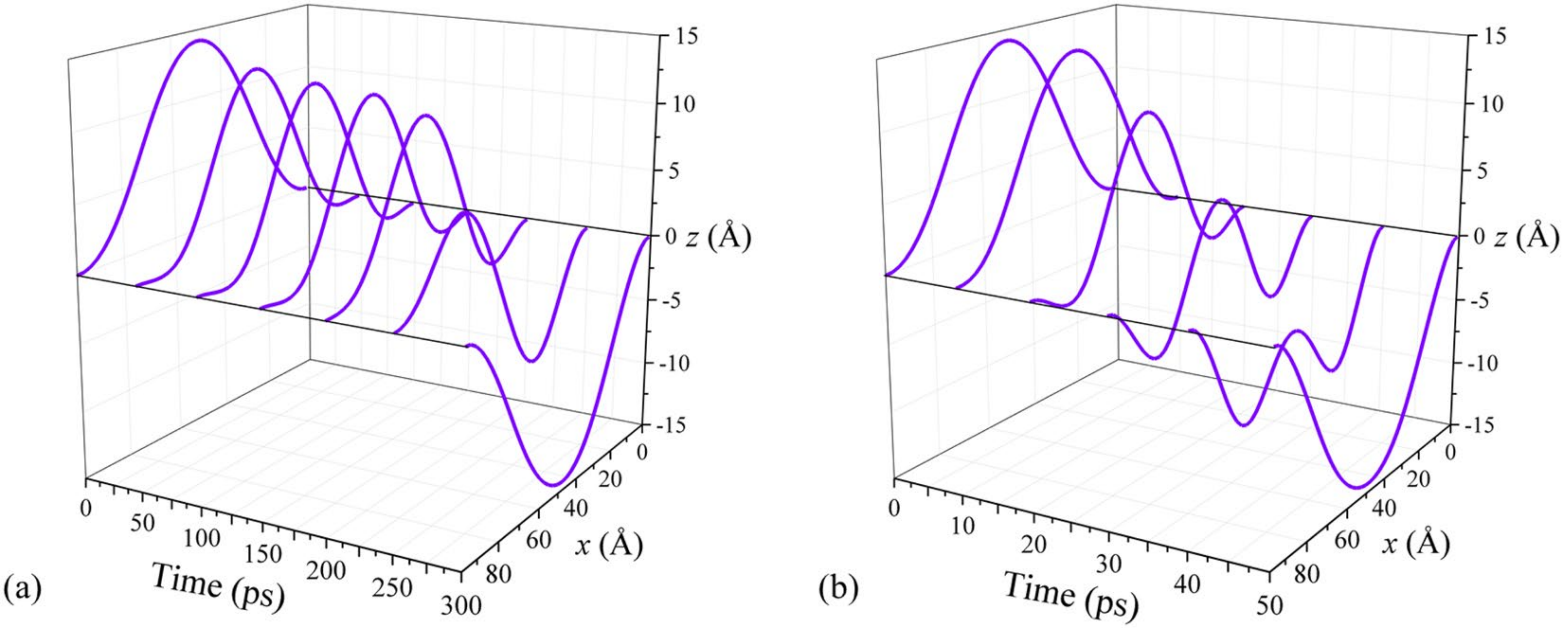
Geometries of membrane *B* in the process of the up-to-down switching at smaller (*F* = 0.5 pN/atom) (**a**) and larger (*F* = 1.49 pN/atom) (**b**) forces. These geometries were obtained using overdamped MD simulations for *d*/*L* = 0.95 (the simulation parameters are provided in the Supplementary Information). The switching occurs through the non-symmetric path at smaller forces (exceeding the threshold) (**a**), and symmetric path at larger forces (**b**).

Depending on the force magnitude, the membrane switching occurs either through the symmetric or non-symmetric membrane profile (see Fig. 3). Figure 3 was obtained using overdamped simulations of membrane dynamics (additional results of these simulations can be found in SI Sec. “MD Simulation Details”). The non-symmetric profile is associated with a smaller energy barrier^14^ and involved in switching by smaller forces. Larger applied forces result in the switching through the symmetric profile. According to our observations and previous work^14^, in all cases, the membrane profile is symmetric at short times. If the force magnitude is sufficient to overcome the energy barrier for the symmetric profile, then, typically, the switching takes place through the symmetric path. Otherwise, a symmetry breaking occurs leading to the switching through the lower energy barrier associated with the non-symmetric membrane profile. Thermal fluctuations help the symmetry breaking.

### Down-to-up transition

It was suggested in ref.^4^ that the memcapacitor membrane can be set into the buckled upwards state 0 (see Fig. 1) by also using the electrostatic attraction between the capacitor plates. When the pulled-down membrane is suddenly released, there are conditions such that the membrane overcomes the potential barrier and ends up in the buckled upwards state. As the kinetic energy plays an important role in the down-to-up transition, this process can not be analyzed using the overdamped dynamics.

**Figure 4:**
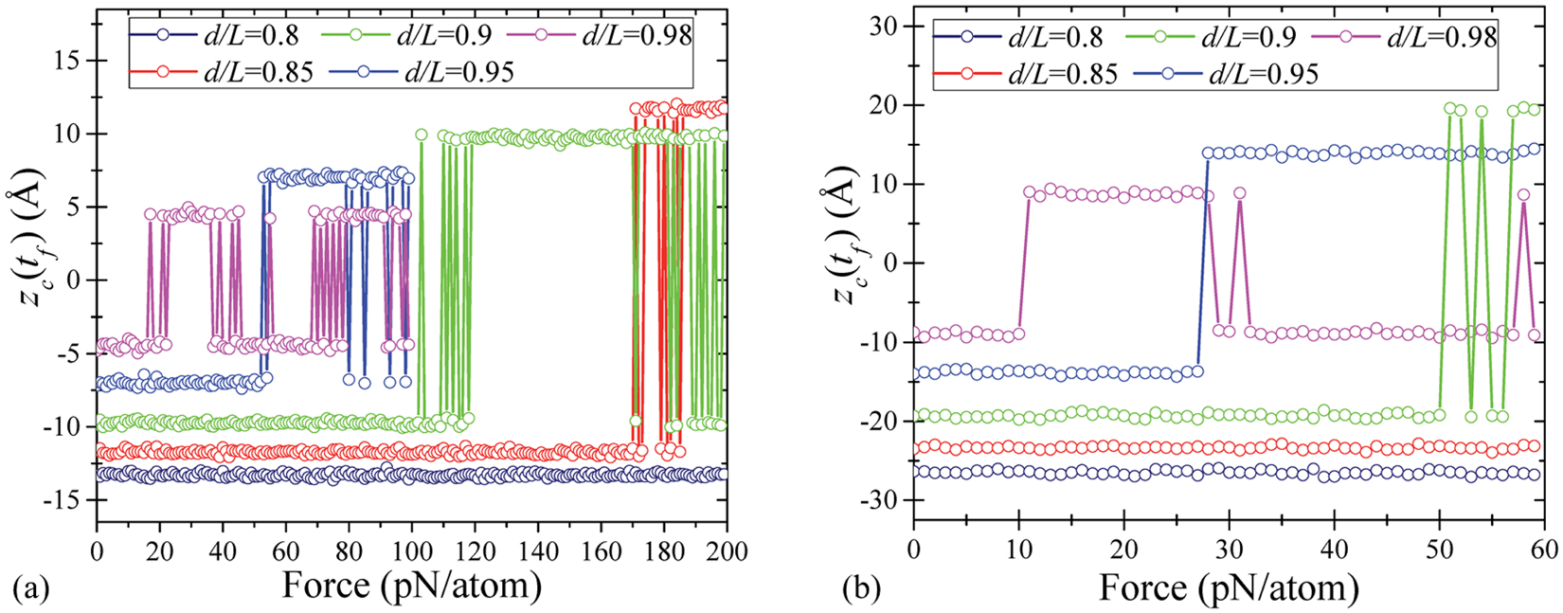
Down-to-up transition: the final position of a central atom of membrane as a function of the applied force magnitude for (**a**) shorter membrane *A* (22 rings length), and (**b**) longer membrane *B* (42 rings length).

In order to simulate the down-to-up transition, every atom of a double-clamped buckled membrane was subjected to a constant force in -*z* direction. After an equilibration period, the forces were removed, and the system was simulated for a time interval sufficient to reach the steady state. The final position of a central atom of membrane is presented in Fig. 4 as a function of the applied force for several values of *d*/*L*.

Figure 4 shows that the threshold forces for the down-to-up transition are larger than those for the up-to-down transition (see Fig. 2). Moreover, the threshold forces for the down-to-up transition are larger for the shorter membrane *A* compared to the longer membrane *B*. It is interesting that the final state of membrane oscillates as a function of the applied force (see *d*/*L* = 0.98 curves in Fig. 4). Qualitatively, having a high kinetic energy, the membrane moves up and down while its energy dissipates. Finally, it gets trapped in one of two stable buckled states.

Overall, our MD results indicate the possibility of writing one bit of information into the state of membrane memcapacitor. In this interpretation, logic 1 corresponds to the membrane buckled downwards, and logic 0 - to the membrane buckled upwards. In addition to 0 → 1 and 1 → 0 transitions presented in Figs 2 and 4, we have verified the occurrence of 1 → 1 and 0 → 0 transitions at the same values of forces required for 0 → 1 and 1 → 0 transitions, respectively. Therefore, logic 0 or 1 can be written into the memcapacitor just by selecting a suitable value of the applied force.

In our work, we considered relatively small nanoribbons, which are mechanically rigid. This is an important requirement for the memcapacitor application as the mechanical nonvolatile information storage is not possible with flexible graphene. From some previous studies^37^ it is known that local structural corrugations (ripples)^38^ disappear in the transition from flexible to rigid graphene. In agreement with this previous work^37^, our molecular dynamics simulations have shown the absence of ripples (spontaneous height fluctuations) in nanoribbons of reported sizes and their existence in larger nanoribbons. The latter, however, are not suitable for the memcapacitor application because of their flexibility. To summarize, while our simulation tools provide a means for ripples detection, we did not observe these in the reported structures that are mechanically rigid.

## Theory of Elasticity

Even though the graphene has a thickness of one atom, the graphene membranes are quantitatively good described by the theory of elasticity^39–42^. This allows us, on one hand, to obtain analytical expressions for the buckled membranes^43–45^ and, on the other hand, perform a comparison with MD simulations.

There is a number of publications dealing with buckled beams and membranes under the transverse load (see, for example, refs^46–49^). Such systems are frequently described in the framework of Euler-Bernoulli theory, which, however, leads to complex analytically unsolvable equations. Bubnov-Galerkin decomposition is a one of the best approaches to find the approximate analytical solution of these equations, although it still requires a complex phase-diagram analysis. In particular, using such analysis, the authors of refs^46–49^ investigated an electrostatically loaded double-clamped membrane above a rigid flat electrode and derived cumbersome conditions for the symmetric snap-through transition, symmetry breaking, existence of bifurcation and pull-in instability.

In this Section, we derive compact but sufficiently precise expressions for the snap-through switching forces based on the theory of elasticity.

### Buckling and snapping-through within the theory of elasticity

#### Description of buckled membranes within the theory of elasticity

Consider a 2D membrane within the theory of elasticity^40,41,50^. The total potential energy of membrane is defined by the deflection *ζ* (along the normal direction *z*) as

3 $$U=\frac{D}{2}\iint {\rm{d}}S{({\rm{\Delta }}\zeta )}^{2}+T\delta S+\iint {\rm{d}}SF\zeta .$$

Here, Δ stands for the 2D Laplacian, *T* = *T*_0_ + *δT* is the total tensile force, *T*_0_ is the force applied by the support and *δT* is the bending tension resulted from the extension,

4 $$\delta T={E}_{2D}\frac{\delta S}{{S}_{0}},\quad \quad \delta S=\iint {\rm{d}}S{(\nabla \zeta )}^{2},$$

*S*_0_ = *wL*, and *F* is the external force density. The compression of membrane corresponds to *T*_0_ < 0.

Given the potential energy, one may also be interested in the dynamics of membrane, which is defined by the equation

5 $$\mu \frac{{\partial }^{2}\zeta }{\partial {t}^{2}}+\mu \gamma \frac{\partial \zeta }{\partial t}-F=-D{\rm{\Delta }}{\rm{\Delta }}\zeta +T\Delta \zeta ,$$

where *μ* is the 2D mass density.

#### Eigenmodes and buckling

The spatial harmonics (eigenmodes) of clamped membranes can be written as

6 $${\zeta }_{n}(x)=\,\cosh ({b}_{n}\frac{x}{d})-\,\cos ({b}_{n}\frac{x}{d})-\frac{\cosh \,{b}_{n}-\,\cos \,{b}_{n}}{\sinh \,{b}_{n}-\,\sin \,{b}_{n}}(\sinh ({b}_{n}\frac{x}{d})-\,\sin ({b}_{n}\frac{x}{d})),$$

where the numbers *b*_*n*_ are the solutions of the equation cosh *b*_*n*_cos *b*_*n*_ = 1. One can find that *b*_*n*_ ≈ 3*π*/2 + *nπ* (in particular, *b*_0_ = 4.73 is close to 3*π*/2 = 4.65). The functions *ζ*_*n*_(*x*) are orthonormal.

Consider a buckled membrane shown in Fig. 1. Its potential energy (Eq. (3)) calculated for the fundamental *n* = 0 mode (taking *ζ*(*x*) = *ζ*_0_(*x*) with *ζ*(*d*/2) ≡ *ζ*_c_) is

7 $$U=-\alpha {\zeta }_{c}^{2}+\beta {\zeta }_{c}^{4}+f{\zeta }_{c},$$

where *α* = *π*^2^*w*(|*T*_0_| − *T*_c_)/(4*d*), *β* = 3*π*^4^*Dw*/(4*ε*^2^*d*^3^), and *f* = *πFdw*/6. Here, *T*_c_ = 4*π*^2^*D*/*d*^2^ is the critical tension. At *α* > 0, that is at |*T*_0_| > *T*_c_, the potential is the double-well potential with minima at symmetric deflections, ${\zeta }_{c}=\pm \sqrt{\alpha \mathrm{/2}\beta }$, and the potential barrier *δU* = *α*^2^/4*β*. (We note that a less realistic case of hinged boundary conditions would lead to simpler expressions for eigenmodes, $\sim \,\sin (\pi nx/d)$, compared to Eq. (6), making the hinged boundary conditions preferable for some calculations. However, this case results in quantitatively different values. For example, the critical tension differs four times.)

We note that the nanoribbon length is given by

8 $$L={\int }_{0}^{d}\text{d}x\sqrt{1+\zeta ^{\prime} 2}.$$

Expanding this expression to the second order, one obtains the interrelation between *L*, *d*, and the maximal deflection *ζ*_c_:

9 $${\zeta }_{{\rm{c}}}\approx 0.64\sqrt[8]{\frac{d}{L}}\sqrt{L(L-d)}.$$

On the other hand, the maximal deflection of buckled membrane can be inferred from the minima of the potential energy, Eq. (7):

10 $${\zeta }_{{\rm{c}}}^{2}=\frac{\alpha }{2\beta }=\frac{2}{{\pi }^{2}}\frac{{L}^{2}}{{E}_{2{\rm{D}}}}(|{T}_{0}|-{T}_{{\rm{c}}}).$$

Equations (9) and (10) link the tensile force *T*_0_ to the parameter *d*/*L*, which can be considered as a measure of deformation of buckled membrane. These equations can be used to express the tensile force *T*_0_ through *d*/*L*.

#### Snap-through transition

Here we present a convenient method to describe the dynamics of membrane subjected to an external force and find the minimal force causing its snap-through transition. The main idea is to expand *ζ*(*x*, *t*) in harmonics *ζ*_*n*_(*x*) (given by Eq. (6)) with amplitudes *q*_*n*_(*t*) as

11 $$\zeta (x,t)=\sum _{n}{q}_{n}(t){\zeta }_{n}(x),$$

and limit the sum to few first terms. We found that in order to describe the symmetric and non-symmetric transitions, it is sufficient to consider *n* = 0,2 and *n* = 0,1 terms in Eq. (11), respectively. The calculation consists in the following. First, we substitute the expansion (11) in Eq. (5). Second, the resulting equation is multiplied by *ζ*_*m*_(*x*) and integrated taking into account the orthogonality of harmonics. Here we emphasize that for the harmonics (6), the integrals are readily calculated, and instead of the integro-differential equation one obtains a system of differential equations for the functions *q*_*n*_(*t*). Inserting the obtained functions *q*_*n*_(*t*) back into Eq. (11) gives us the description of membrane dynamics. In the following, the results of these calculations (performed using the time normalized by the membrane characteristic frequency ${\omega }_{{\rm{c}}}=\mathrm{(1/}{L}^{2})\sqrt{D/\mu }$ and *t*_c_ = 1/*ω*_c_)^51^ are analyzed for membrane *B*.

**Figure 5:**
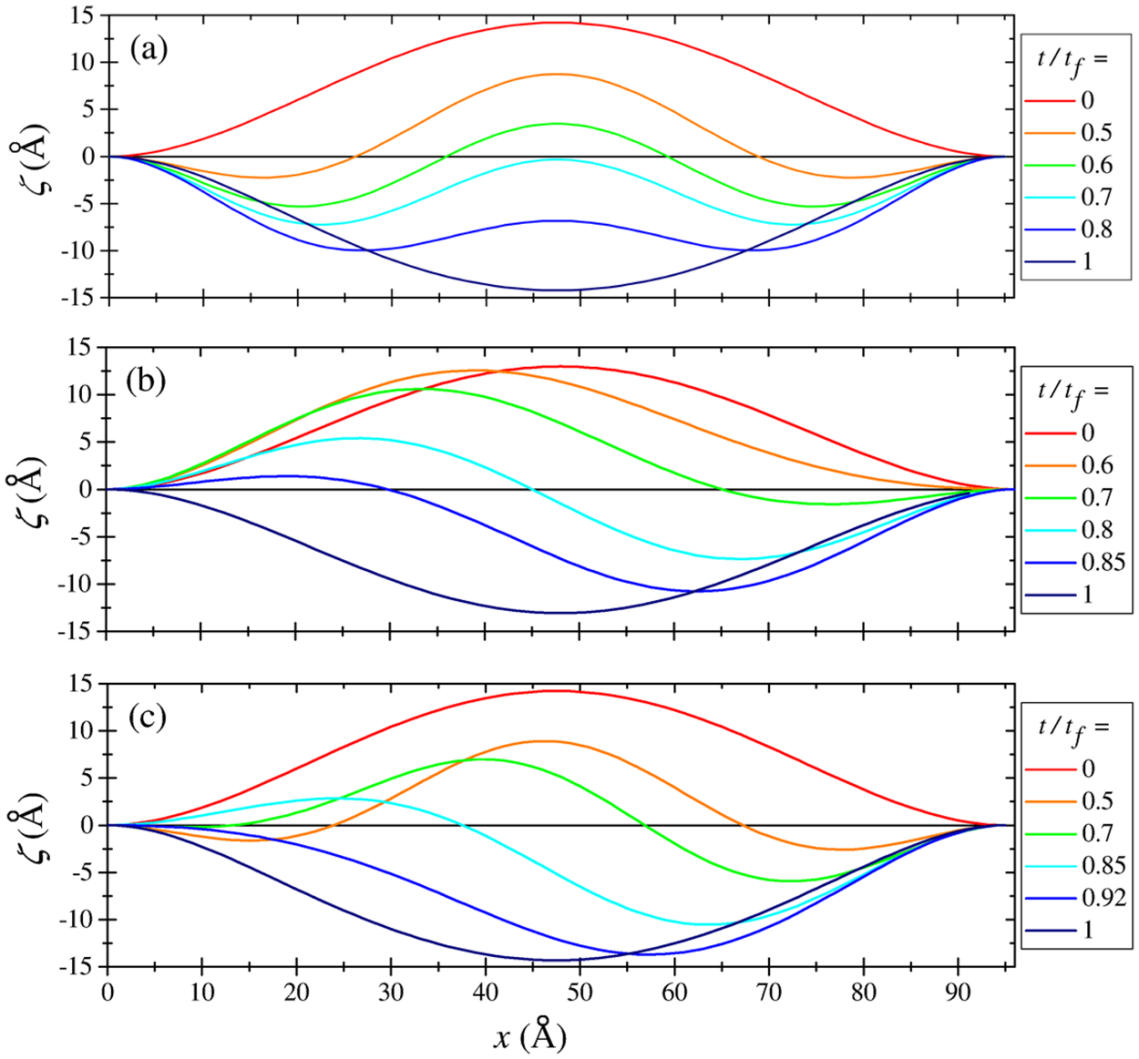
Up-to-down transition within the theory of elasticity. (**a**) Fully symmetric switching. (**b**) Non-symmetric switching caused by a small asymmetry in the applied force. (**c**) More realistic switching scenario based on a symmetric force, where the initial symmetric distortion becomes non-symmetric at longer times. For all panels *d*/*L* = 0.95.

Consider the results presented in Fig. 5. Figure 5(a) demonstrates the case of fully symmetric switching, when the dynamics can be described by *n* = 0 and 2 harmonics. Next, we introduce a small asymmetry into the problem via a small non-symmetric modification of the force (of the order of 0.1%). This results in the non-symmetric dynamics of Fig. 5(b), which can be described in terms of *n* = 0 and 1 harmonics. We note that the force needed for the non-symmetric snap-through transition is smaller than that for the symmetric one, which will also be analyzed below in more detail. Finally, in Fig. 5(c) we illustrate a combination of the above regimes found using a symmetric force, when a tiny fluctuation in the numerical solution changes the symmetric dynamics to the non-symmetric one. Note that this case is close to the one discussed in ref.^14^.

**Figure 6:**
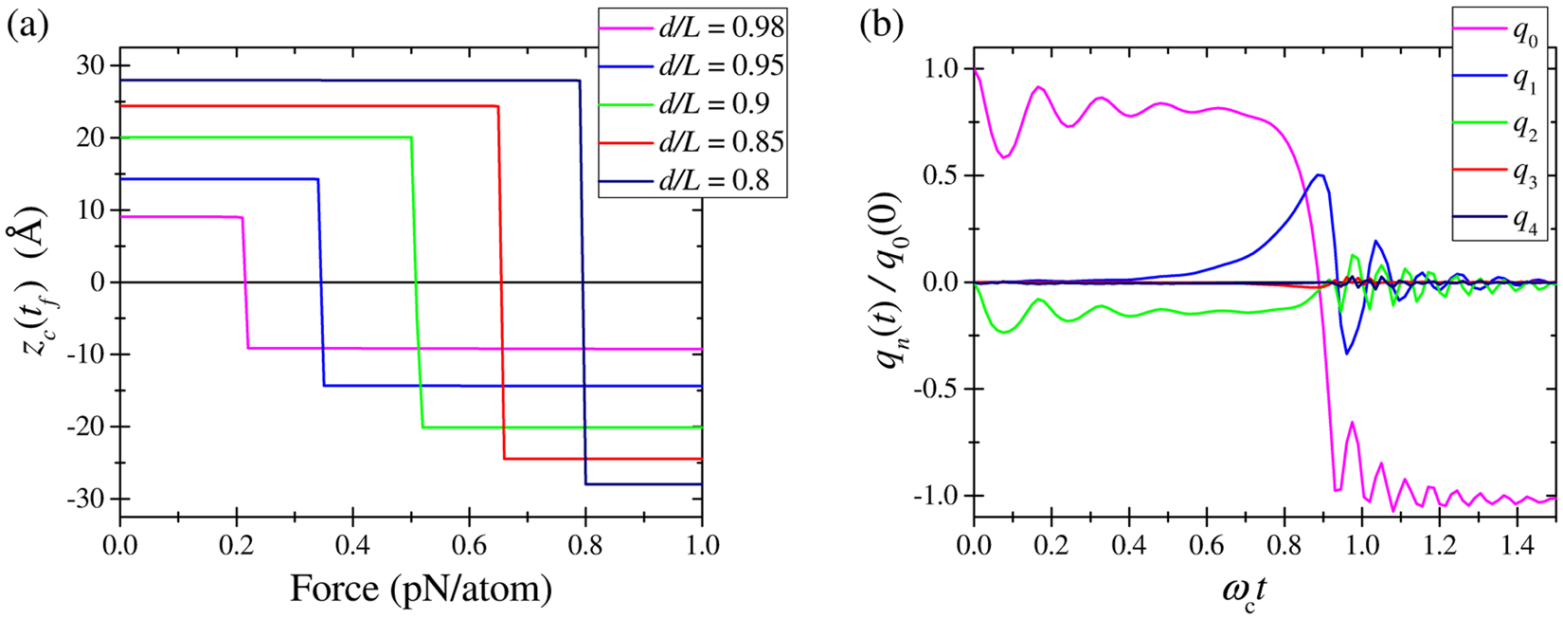
Numerical simulation of up-to-down transition. (**a**) The final central-point deflection *ζ*_c_ versus the applied transverse force *F* for several values of *d*/*L*. (**b**) Time-dependence of the first few harmonics’ amplitudes *q*_*n*_(*t*).

Figure 6(a) depicts the dependence of the final position of membrane center, *ζ*_c_(*t*_*f*_), on the applied force *F*. At a certain value of force, *F* = *F*^↓^, the membrane switches from the buckled upwards state to the buckled downwards state. Our calculations show that the threshold force is proportional to the initial central-point deflection *ζ*_c_ and is different for the symmetric (s) and non-symmetric (ns) dynamics. Namely, this force, being multiplied by the membrane area *wL*, reads

12 $$\frac{{F}_{s,\mathrm{ns}}^{\downarrow }}{{F}_{0}}={C}_{s,\mathrm{ns}}\sqrt{2.44}\frac{{\zeta }_{{\rm{c}}}}{d}={C}_{s,\mathrm{ns}}{(\frac{L}{d})}^{3/8}\sqrt{\frac{L}{d}-1},$$

13 $${F}_{0}=\frac{Dw}{{L}^{2}},\quad {C}_{{\rm{ns}}}=\mathrm{253.4,}\quad {C}_{{\rm{s}}}=\mathrm{359.1.}$$

Since the non-symmetric transition is energetically favorable, we expect that this value of force is the one to be used in the device design/experiment analysis. We note that estimations based on Eq. (12) are in a good agreement with results found by other methods as we discuss later and illustrate in Sec. “Discussion” below. Figure 6(b) presents the time-dependence of harmonics’ amplitudes *q*_*n*_(*t*) in the up-to-down transition. Importantly, the dynamics is well described by *n* = 0, 1, and 2 harmonics, while *n* = 3 and 4 harmonics amplitudes are negligibly small.

Our calculations (similar to the consideration of the up-to-down force above) demonstrate that the minimal force needed for the down-to-up transition is proportional to ${\zeta }_{{\rm{c}}}^{2}$ as

14 $$\frac{{F}^{\uparrow }}{{F}_{0}}={C}^{\uparrow }2.44{(\frac{{\zeta }_{{\rm{c}}}}{d})}^{2}={C}^{\uparrow }{(\frac{L}{d})}^{\mathrm{3/4}}(\frac{L}{d}-1),$$

15 $${C}^{\uparrow }=8.48\cdot {10}^{4}\mathrm{.}$$

See also SI Subsec. “Down-to-up transition”, where the down-to-up transition is illustrated graphically.

### Phenomenological elasticity theory

In this Section, in order to avoid all complications associated with the Bubnov-Galerkin decomposition, we elaborate a different approach describing the up-to-down and down-to-up transitions in a compact analytical form.

#### Ansatz

Following our observation of the existence of the symmetric and non-symmetric membrane profiles in the snap-through transition (see Fig. 3), we introduce two simplest polynomial functions to describe such symmetric (s) and non-symmetric (ns) shapes of membrane:

16 $${\theta }_{{\rm{s}}}(s)={A}_{{\rm{s}}}({s}^{2}-\frac{1}{4})s({s}^{2}-{c}_{0}^{2}),$$

17 $${\theta }_{{\rm{ns}}}(s)={A}_{{\rm{ns}}}({s}^{2}-\frac{1}{4})(s-{c}_{1})(s-{c}_{2})\mathrm{.}$$

Here *θ*_*i*_(*s*) is the angle that the membrane makes with the horizontal, *s* is the internal coordinate that changes between −1/2 and 1/2, *A*_*i*_ and *c*_*i*_ are coefficients, and *i* = {s, ns}. Clearly, Eqs (16) and (17) describe the double-clamped membrane as *θ*_*i*_(±1/2) = 0.

#### Up-to-down transition

Our goal here is to estimate the minimal force required for the snap-through transition. In this subsection, we assume that the snap-through transition is induced by a slowly increasing force such that the system always stays in the potential energy minimum. At zero applied force, there are two minima of the potential energy corresponding to the two stable states of membrane. The applied force modifies the potential energy landscape such that, starting at a certain force, the potential energy has a single minimum. Here, this value of force is found and considered as an estimation for the threshold switching force.

In the presence of a constant force *F* in the downward direction, the membrane potential energy is given by

18 $$U={U}_{b}+{U}_{str}+{U}_{ext}=\frac{Dw}{2L}{\int }_{-\mathrm{1/2}}^{\mathrm{1/2}}{[\frac{\partial \theta }{\partial s}]}^{2}{\rm{d}}s+{U}_{str}+{U}_{ext},$$

where *U*_*b*_ is the bending energy, *U*_*str*_ is the stretching energy, *U*_*ext*_ is the potential energy due to the applied force. Then, using *U*_*ext*_ = *Fz*_*cm*_, where *z*_*cm*_ is *z* coordinate of the center of mass, and neglecting *U*_*str*_ term not important for the up-to-down transition (see, e.g., Fig. S1 that indicates the insignificance of *U*_*str*_ in buckled membrane), we get the following equation for the potential energy extrema:

19 $$F=-\frac{{\rm{d}}{U}_{b}}{{\rm{d}}{z}_{cm}}=-\frac{{\rm{d}}{U}_{b}}{{\rm{d}}{c}_{i}}{(\frac{{\rm{d}}{z}_{cm}}{{\rm{d}}{c}_{i}})}^{-1},$$

where *i* = {0, 1} corresponds to {s, ns}, respectively. Equation (19) allows finding *c*_0_ and *c*_1_ for a given strength of the applied force.

The minimal force needed for the snap-through transition corresponds to the maximum of *F*(*z*_*cm*_) and, for the transition through the symmetric shape, is given by

20 $${F}_{{\rm{s}}}^{\downarrow }=440\frac{Dw}{{L}^{2}}\sqrt{\frac{L-d}{L}}$$

taking place at *c*_0_ = 0.3589 (the corresponding membrane profile is presented in Fig. S4). At this value of *c*_0_, the bending energy is *U*_*b*,s_ = 108*Dw*(*L* − *d*)/*L*^2^. Performing the same calculations for the non-symmetric shape, one can find that the force needed to support the equilibrium non-symmetric shape decreases with the progress of switching. A zero force is reached at ${c}_{1}=-{c}_{2}=\mathrm{1/}(2\sqrt{5})$ that corresponds to the maximum of *U*_*b*,ns_ = 90*Dw*(*L* − *d*)/*L*^2^. A possible (rough) estimation for the threshold switching force can be obtained taking the limit *c*_1_ → ∞ leading to

21 $${F}_{ns}^{\downarrow }=281\frac{Dw}{{L}^{2}}\sqrt{\frac{L-d}{L}}$$

and *U*_*b*,ns_ = 42*Dw*(*L* − *d*)/*L*^2^.

In fact, a realistic scenario of the switching through the non-symmetric shape can be imaged as follows. We start with a symmetric membrane at *F* = 0 and slowly increase the force. The symmetry breaking occurs at a certain value of force, say, *F*^↓^. The dynamics of switching is a complex process significantly relying on thermal fluctuations. As the switching dynamics can not be reached within the framework of our model, for estimation purposes, we assume that *F*^↓^ corresponds to the point where the bending energies and applied forces for the symmetric and non-symmetric shapes are the same. One can find that both conditions are satisfied at *c*_0_ = 0.4683 and *c*_1_ = 0.6948, so that *U*_*b*_ = 47.8*Dw*(*L* − *d*)/*L*^2^ and

22 $${F}^{\downarrow }=263.53\frac{Dw}{{L}^{2}}\sqrt{\frac{L-d}{L}}\mathrm{.}$$

Consequently, at *F* < *F*^↓^, the membrane keeps the symmetric shape and switches to the non-symmetric one as soon as *F* reaches *F*^↓^. As there is no barrier involved in the non-symmetric switching, no further increase in the applied force is required to complete the snap-through transition through the non-symmetric shape.

#### Down-to-up transition

As the stretching energy *U*_*str*_ (see Eq. (18)) plays an important role in the down-to-up transition, it needs to be taken into account. For our purposes, it is sufficient to approximate *U*_*str*_ as

23 $${U}_{str}=\frac{{E}_{2D}w}{2L}{\rm{\Delta }}{L}^{2},$$

where Δ*L* is the change in the membrane length. In the approximation of small elongation, ${\rm{\Delta }}L\ll L$, one can assume that both *U*_*b*_ and the shape of the stretched membrane are not significantly modified compared to *F* = 0 case. In this situation, at the threshold of transition, the stretching energy (Eq. (23)) is equal to the difference between the maximal and minimal bending energies (the energy conservation condition). Using the energies given below Eq. (S8) we find

24 $$\frac{{E}_{2D}w}{2L}{\rm{\Delta }}{L}^{2}={U}_{b}^{max}-{U}_{b}^{min}=161Dw\frac{L-d}{{L}^{2}}\mathrm{.}$$

Moreover, at equilibrium

25 $$F({z}_{cm})=\frac{{E}_{2D}w}{2L}\cdot \frac{{\rm{d}}({\rm{\Delta }}{L}^{2})}{{\rm{d}}{z}_{cm}}\mathrm{.}$$

Under the condition of small ${\rm{\Delta }}L\ll L$, the center of mass position of membrane (buckled downwards) can be expressed as

26 $${{z}_{cm}}^{\ast }=-0.3187\sqrt{L(L-d)}+\frac{{\rm{d}}{z}_{cm}}{{\rm{d}}({\rm{\Delta }}L)}{\rm{\Delta }}L,$$

with

27 $$\frac{{\rm{d}}{z}_{cm}}{{\rm{d}}({\rm{\Delta }}L)}=0.3187\frac{2L-d}{2\sqrt{L(L-d)}}\mathrm{.}$$

Using Eqs (24) and (27), Eq. (25) can be rewritten as

28 $${F}^{\uparrow }=79.62\sqrt{2D{E}_{2D}}\frac{w}{L}\frac{L-d}{2L-d}\mathrm{.}$$

We have verified that the threshold switching force for the down-to-up transition given by Eq. (28) is in agreement with our typical MD simulations results. A very good agreement was obtained with MD simulations performed with very small energy dissipations.

## Density Functional Theory

A DFT investigation of the up-to-down transition was performed following the previously reported study of the up-to-down switching of buckled graphene membrane^14^. In the present calculations, we focused on the effects of boundary conditions and buckling strength on the up-to-down threshold switching force. DFT computational details can be found in SI Sec. “DFT Calculations”.

**Figure 7:**
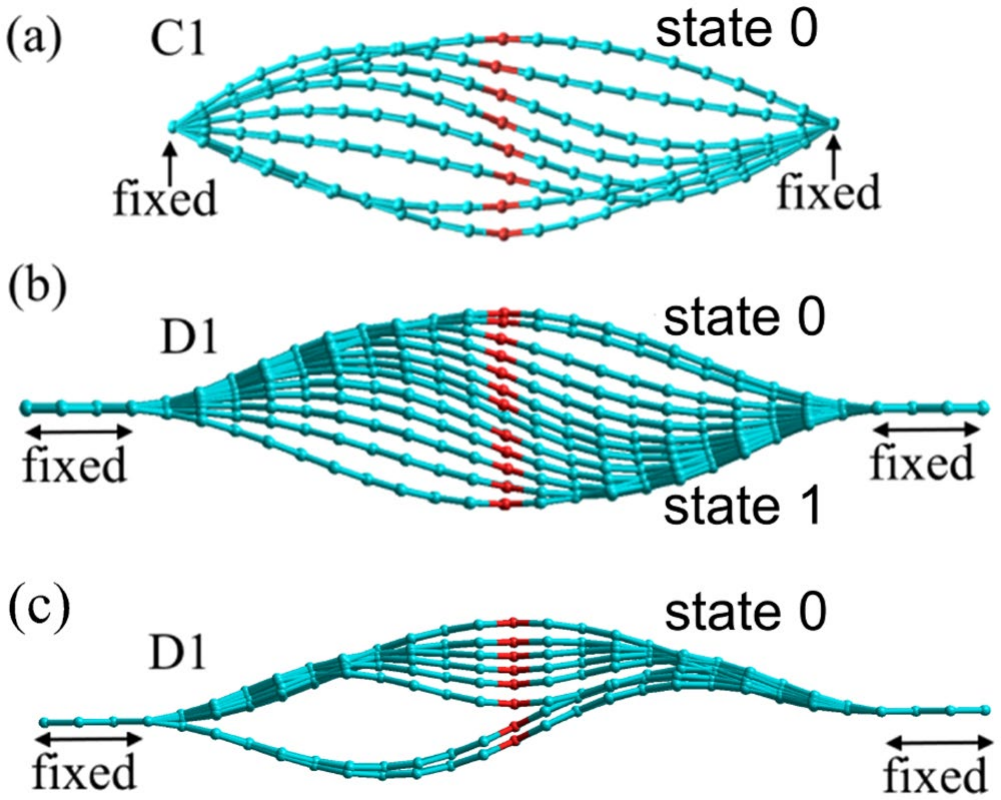
Geometries of membranes C1 (hinged boundary conditions, (**a**)) and D1 (clamped boundary, (**b**,**c**)) in the process of the up-to-down switching. The switching occurs through non-symmetric paths (**a**,**b**), and initially symmetric path (**c**). These geometries were found using DFT optimization of geometries obtained utilizing a progressive displacement of central atoms.

We investigated a buckled zigzag graphene nanoribbon specified in SI. Two types of boundary conditions were examined: (1) hinged boundary conditions (configurations C1 with *d*/*L* = 0.96, Fig. 7(a), C2 with *d*/*L* = 0.78, and C3 with *d*/*L* = 0.63), and (2) clamped boundary conditions (configuration D1 with *d*/*L* = 0.96, Fig. 7(b,c)). The initial geometries of nanoribbons corresponded to the buckled upwards state. In order to simulate the up-to-down transition, we used the approach developed in ref.^14^. Specifically, we performed a series of DFT calculations with the central chain of membrane atoms (marked red in Fig. 7) gradually displaced in −*z* direction from its position in the buckled upwards state. The membrane geometry found at the preceding deformation step was used to build its subsequent modification. In contrast to ref.^14^, in the present calculations only *z* coordinates of the central chain of atoms were constrained.

The deformation energies of buckled graphene are reported in SI. Our force estimation shows that the threshold switching force for the up-to-down transition is about 3.8 pN/atom for C1, 11.1 pN/atom for C2, and 16.4 pN/atom for C3. Moreover, the non-symmetric switching force for D1 is 3.7 pN/atom and the symmetric one is about 11.5 pN/atom. As the non-symmetric switching forces for D1 and C1 are close to each other, we use the results for C1-C3 for the order-of-magnitude comparison with MD and elasticity theory results.

## Discussion

### Four methods to describe the snap-through transitions

**Figure 8:**
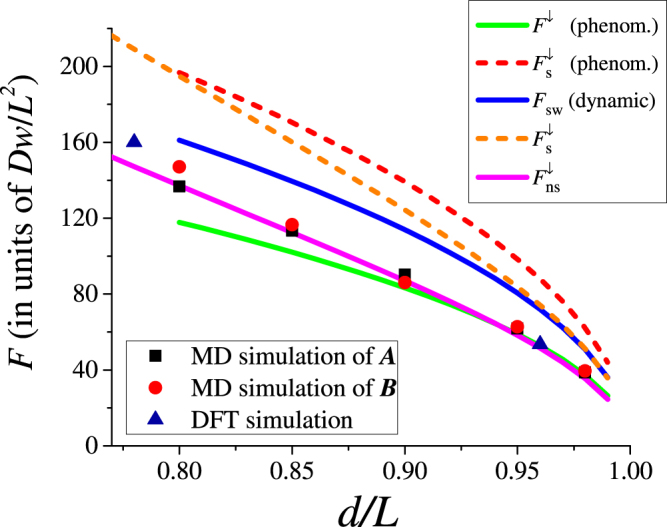
Comparison between the up-to-down threshold forces, calculated by four different approaches. Green, red, and blue lines were calculated in the non-symmetric, adiabatic and dynamic symmetric switching regimes, respectively. The results of our MD simulations are shown with squares for nanoribbon *A* and circles for *B*. The forces calculated by means of the elasticity theory, Eq. (12), are shown with the magenta solid line for the non-symmetric snap-through and with the orange dashed line for the symmetric transition. The DFT results are presented by triangles.

As the key finding, Fig. 8 presents the dependence of the up-to-down threshold switching force (calculated with different approaches) on the membrane deformation parameter *d*/*L*. In particular, this figure demonstrates that the threshold forces obtained by a number of different methods are in good agreement with each other.

We emphasize that:

(i) MD is widely used approach to describe the dynamics of nanoscale systems. In our work, MD clearly shows the possibility of 0 → 1 and 1 → 0 transitions of graphene membrane. We have also verified the occurrence of 1 → 1 and 0 → 0 transitions at the same values of forces required for 0 → 1 and 1 → 0 transitions, respectively.
(ii) In the framework of elasticity theory, the membrane is described by means of an integro-differential equation for the deflection *ζ*(*x*, *t*). Expanding the function *ζ*(*x*, *t*) into membrane’s harmonics allows reducing the integro-differential equation to a system of differential equations. This approach was utilized in Subsec. “Buckling and snapping-through within the theory of elasticity”. There it was shown that the up-to-down threshold switching force depends linearly on the initial central-point deflection, *F*^↓^ ∝ *ζ*_c_, while the down-to-up snap-through force at low dissipation depends quadratically on the initial deflection, ${F}^{\uparrow }\propto {\zeta }_{{\rm{c}}}^{2}$. Besides, we have demonstrated that with a high accuracy, the relevant membrane’s dynamics can be described (and visualized) just by two harmonics.
(iii) The phenomenological approach based on the theory of elasticity has allowed us to obtain analytical expressions for the up-to-down and down-to-up switching forces for buckled graphene membrane. The forces for 0 → 1 and 1 → 0 transitions are in a good agreement with MD simulations results.
(iv) DFT is a non-standard method for studying mechanical properties of membranes. Similarly to other methods, DFT has shown that the non-symmetric switching pathway is the preferable one. This method also provides a consistent estimation for the up-to-down threshold switching force.

### Implications for memcapacitor design

From the application point of view, the membrane memcapacitor should have a strong ‘memory content’, namely, the device characteristics in its two logic states should be sufficiently different so as to provide a significant influence on other elements of electronic circuits^52^. For this purpose, it is desirable that both the distance between the fixed edge of membrane and second electrode (*h* in Fig. 1) and the maximal deflection of membrane (*z*_*s*_(0)) are chosen (much) smaller than the membrane length. Moreover, the gap between electrodes should be larger than the maximal membrane deflection, *h* > *z*_*s*_(0) (see Eq. (S2) for the definition of *z*_*i*_(*s*)). For given capacitances *C*_0_ and *C*_1_ of the states 0 and 1 (logic 1 corresponds to the membrane buckled downwards, and logic 0 - to the membrane buckled upwards) such that *C*_1_/*C*_0_ < 3.01, one can find that

29 $$h=0.3210\sqrt{L(L-d)}\frac{{C}_{1}+{C}_{0}}{{C}_{1}-{C}_{0}}\mathrm{.}$$

Next, we need an expression for the force applied to the membrane expressed through the input variable of memcapacitor such as the applied voltage *V* (see Eqs (1) and (2)). Since the energy of the plate can be written as

30 $${U}_{ext}({z}_{cm})=-\frac{{\varepsilon }_{0}{V}^{2}A}{\mathrm{2(}h+{z}_{cm})},$$

where *ε*_0_ is the vacuum permittivity and *A* = *d* · *w* is the plate area, the force is given by

31 $$F({z}_{cm})=-\frac{{\varepsilon }_{0}{V}^{2}A}{\mathrm{2(}h+{z}_{cm}{)}^{2}}\mathrm{.}$$

**Figure 9:**
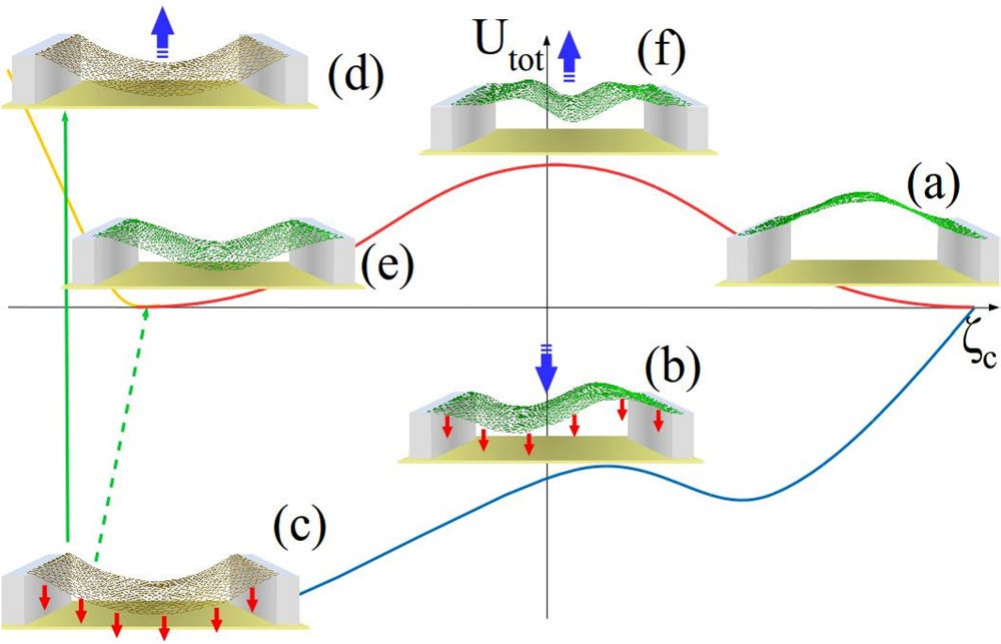
Schematic representation of transition pathways in the switching of membrane memcapacitor (red arrows represent the applied electrostatic force, bold blue arrows represent the movement of membrane). The buckled upwards membrane (structure (**a**)) evolves into the buckled downwards state (**c**) through a non-symmetric transition state (**b**). Note that in the presence of force, (**c**) is also a transition state. After a slow voltage removal (dashed green arrow), the membrane remains in the buckled downwards state (**e**) (the up-to-down transition). In case of fast force removal, the membrane does not have enough time to relax its tension (**d**) and start moving into the upward state (**a**) through a symmetric transition state (**f**) (the down-to-up transition).

The overall scheme of memcapacitor switching is schematically presented in Fig. 9. Let us consider first the up-to-down transition (path (a–c,f) in Fig. 9). As mentioned in SI Sec. “Phenomenological Elasticity Theory”, the smallest threshold force for this transition is associated with the switching through the non-symmetric г state and corresponds to ${c}_{1}^{\ast }=0.6948$. In this way, the buckled upwards membrane (structure (a) in Fig. 9) evolves into the buckled downwards state according to (b) and (c) in Fig. 9. After the voltage removal, the membrane remains in the buckled downwards state ((e) in Fig. 9). In order to estimate the minimal (threshold) voltage required for this transition, Eq. (22) is rewritten accounting for Eq. (31):

32 $$\frac{{\varepsilon }_{0}{V}^{2}dw}{\mathrm{2(}h+{z}_{cm}({c}_{1}^{\ast }{))}^{2}}=263.53\frac{Dw}{{L}^{2}}\sqrt{\frac{L-d}{L}}\mathrm{.}$$

Consequently,

33 $${V}_{0\to 1}=22.96\frac{h+{z}_{cm}({c}_{1}^{\ast })}{L}\sqrt{\frac{D}{{\varepsilon }_{0}\cdot d}\sqrt{\frac{L-d}{L}}},$$

where ${z}_{cm}({c}_{1}^{\ast })=0.3203\sqrt{L(L-d)}$. Eq. (33) provides an easy way to find the threshold voltage necessary for the up-to-down transition of membrane.

In the down-to-up transition, the initial buckled downwards membrane ((e) in Fig. 9) is first stretched by the applied force ((c) in Fig. 9). When the force is removed, the membrane state becomes (d). If the potential energy of (d) is sufficient to overcome the potential barrier height symmetrically (f), then the membrane may end up in the buckled upwards state (a). These general processes are partially described by Eqs (24) and (25). Because of the membrane elongation in (c,d), a refined value of *z*_*cm*_ should be used in relevant calculations.

The threshold voltage can be estimated based on the energy conservation (Eq. (24)) and force equilibrium (Eq. (25)) conditions accounting for ${z}_{cm}^{\ast }$ from Eqs (26) and (27). One can find that

34 $$\frac{{\varepsilon }_{0}{V}^{2}d\cdot w}{\mathrm{2(}h+{z}_{cm}^{\ast }{)}^{2}}=79.62\sqrt{2D{E}_{2D}}\frac{w}{L}\frac{(L-d)}{\mathrm{(2}L-d)}$$

and

35 $${V}_{1\to 0}=\mathrm{12.62(}h+{z}_{cm}^{\ast })\sqrt{\frac{\sqrt{2D{E}_{2D}}}{{\varepsilon }_{0}\cdot d\cdot L}\frac{(L-d)}{\mathrm{(2}L-d)}}\mathrm{.}$$

An additional aspect of membrane dynamics – an estimation of thermally-induced switching time – is considered in SI Sec. “Stability of Buckled Membrane”.

## Concluding Remarks

We have investigated the snap-through transition of a buckled graphene membrane using a variety of computational and theoretical tools. The main results of this paper are the expressions for the threshold switching forces (Eqs (12 and 22) for the up-to-down transition and Eqs (14 and 28) for the down-to-up one) and corresponding voltages (Eqs (33) and (35)). Our analytical results are supported by the results of numerical simulations, MD and DFT calculations. We expect that our findings will find applications in the design, fabrication and analysis of membrane-based memory devices.

## Electronic supplementary material

supplementary information
