# Supplementary material for: Snap-through transition of buckled graphene membranes for memcapacitor applications
Source: Sci Rep. 2018 Feb 23;8:3566. doi: 10.1038/s41598-018-21205-3 (PMC5824796; doi:10.1038/s41598-018-21205-3)
Supplement: Supplementary file 1 — supplementary information [file 41598_2018_21205_MOESM1_ESM.pdf]

Supplementary Information for: Snap-through transition of buckled graphene membranes for memcapacitor applications

Ruslan D. Yamaletdinov[1,2,*], **Oleg V. Ivakhnenko**[3,4], **Olga V. Sedelnikova**[1,2], **Sergey N. Shevchenko**[3,4], **and Yuriy V. Pershin**[1,5,†]

[1]Nikolaev Institute of Inorganic Chemistry SB RAS, Novosibirsk 630090, Russia
[2]Novosibirsk State University, Novosibirsk 630090, Russia
[3]B. I. Verkin Institute for Low Temperature Physics and Engineering, Kharkov 61103, Ukraine
[4]V. N. Karazin Kharkov National University, Kharkov 61022, Ukraine
[5]Department of Physics and Astronomy, University of South Carolina, Columbia, South Carolina 29208, USA
[*]yamaletdinov@niic.nsc.ru
[†]pershin@physics.sc.edu

MD Simulation Details

We used NAMD2 (see Ref. [1]) – a parallel classical molecular dynamics package – to simulate the dynamics of buckled graphene nanoribbons subjected to external force. NAMD was developed by the Theoretical and Computational Biophysics Group in the Beckman Institute for Advanced Science and Technology at the University of Illinois at Urbana-Champaign.

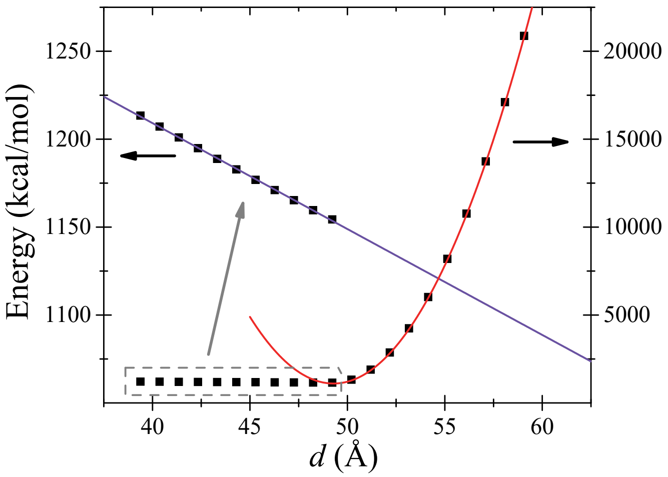

Figure S1. Calculated energies of a stretched and compressed graphene nanoribbon of the free length of $L = 49.2$ Å and width of $w = 40$ Å as a function of the distance d between its fixed edges. This calculation was performed to verify the selected force-field parameters of carbon atoms in graphene. The fitting curves are represented by lines and are based on $E_{2D} = 342$ N/m and $D = 1.6$ eV parameters values. The calculation was performed using hinged boundary conditions.

In graphene, the interactions between the carbon atoms were described using the standard 2-body spring bond, 3-body angular bond (including the Urey-Bradley term), 4-body torsion angle and Lennard-Jones potential energy terms[2]. The interaction constants were optimized in order to fit the experimentally observed properties of graphene. The known values were selected for the equilibrium angles. The Lennard-Jones coefficients were chosen to match the AB stacking distance and energy of graphite[3]. A global optimization was performed over the remaining parameters to match the in-plane stiffness ($E_{2D} = 342$ N/m), bending rigidity ($D = 1.6$ eV) and equilibrium bond length ($a = 1.421$ Å) of graphene. We performed a series of test calculations that have demonstrated that the in-plane stiffness and bending rigidity of a selected graphene nanoribbon are in perfect agreement with the listed above values (see Fig. S1).

A 1 fs time step was used and the system temperature is kept at room temperature with a Langevin damping parameter of $\gamma = 0.2$ ps^{-1} in the equations of motion. The van der Waals interactions were gradually cut off starting at 10 Å from the atom until reaching zero interaction 12 Å away. In each simulation, the buckled membrane was used as an initial condition. Typically, the system was equilibrated for 100 ps in the presence of the applied force and its subsequent dynamics (after the force removal) was typically simulated for 100 ps (these times were changed to 500 ps in the case of overdamped simulations). The final state was determined according to z-coordinate of an atom in the cental part of the membrane.

We also performed a series of overdamped simulations of membrane dynamics. This type of simulations was made using zero temperature and strong damping that significantly suppress the kinetic energy of the membrane. As a result, we have obtained more regular final states of the membrane (see Fig. S2) and slightly different values of the threshold switching force, which, however, can be more easily interpreted as now the kinetic energy can be disregarded. Comparing Fig. 2(b) and Fig. S2 we note that the overdamped calculations provide somewhat larger/comparable estimations for the threshold switching force.

Elasticity Theory

Down-to-up transition

A study of the down-to-up transition based on the standard elasticity theory is presented in Fig. S3. Fig. S3(a) shows the final position of membrane depending on the applied force magnitude. In these calculations, the force was applied for a finite interval of time and removed at $\omega_c t = 0.6$. It was observed that the final state of membrane depends on few modeling parameters (the applied force, damping rate, etc.). The possibility of the down-to-up transition is clearly seen in Fig. S3(a). The time-dependence of harmonics amplitudes is exemplified in Fig. S3(b) for a particular value of force. We note that in Fig. S3(b)

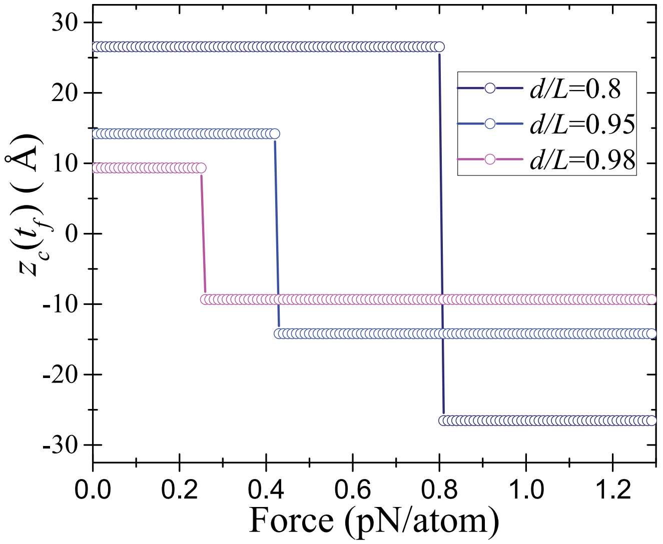

Figure S2. Up-to-down transition: the final position of a central atom of membrane as a function of the applied force magnitude in an overdamped calculation ($T = 0$, $\gamma = 1$ ps^{-1}) for membrane $\boldsymbol{B}$ (42 rings length).

the amplitudes q_n are normalized to q_0 at $t = 0$. Fig. S3(b) shows that the harmonics amplitudes are modified by the applied force (e.g., it is clearly seen that $|q_0(t)/q_0(0)| > 1$ at $\omega_c t < 0.6$). Importantly, the amplitudes of higher harmonics are small and can be neglected.

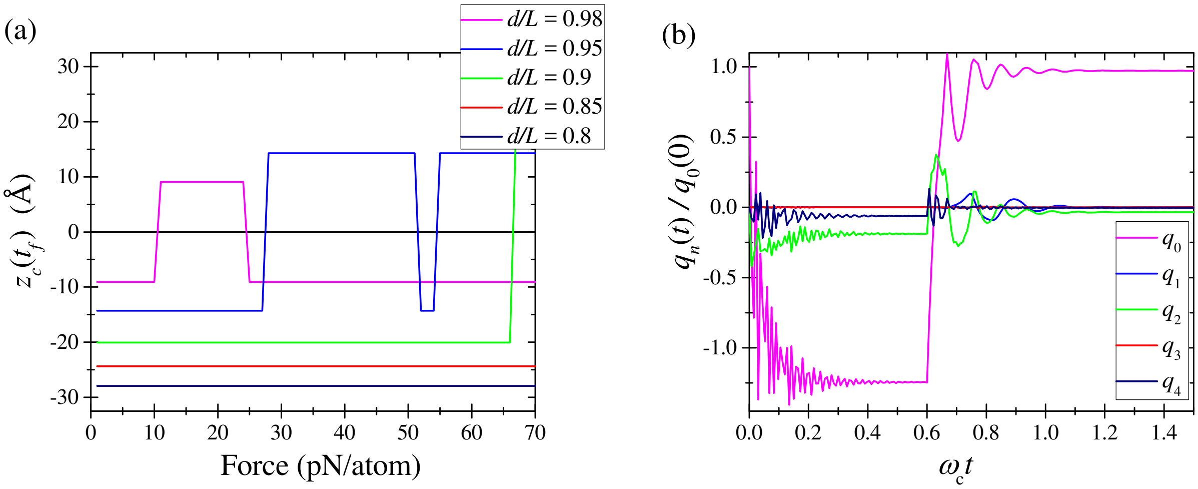

Figure S3. Numerical simulation of the down-to-up transition. (a) The central-point deflection ζ_c versus the applied transverse force F for several values of d/L. Snapping-through appears at the critical force value $F = F^{\uparrow}$. (b) Time-dependence of the first few harmonics amplitudes $q_n(t)$.

Phenomenological elasticity theory

The Cartesian coordinates, which give the position of a membrane element defined by the internal coordinate s, are related to the angle θ_i (see Eqs. (16) and (17)) as

$$x_i(s) = L \int_{-1/2}^{s} \cos\theta_i(s')\mathrm{d}s', \tag{S1}$$

$$\zeta_i(s) = L \int_{-1/2}^{s} \sin\theta_i(s')\mathrm{d}s'. \tag{S2}$$

According to the above equations, $x_i(-1/2) = 0$ and $\zeta_i(-1/2) = 0$. Another pair of boundary conditions is given by

$$x_i(1/2) = d, \qquad \zeta_i(1/2) = 0. \tag{S3}$$

In the limit of small deflections, the boundary conditions, Eq. (S3), can be resolved for the coefficients A_i and c_i in Eqs. (16) and (17):

$$A_\mathrm{s} = \pm\frac{48\sqrt{385}\sqrt{1-\frac{d}{L}}}{\sqrt{5-88c_0^2+528c_0^4}}, \tag{S4}$$

$$c_2 = -\frac{1}{20}c_1, \tag{S5}$$

$$A_\mathrm{ns} = \pm\frac{c_1 240\sqrt{35}\sqrt{1-\frac{d}{L}}}{\sqrt{1200c_1^4-56c_1^2+3}}. \tag{S6}$$

Therefore, the value of a single parameter c_0 (or c_1) defines completely the symmetric (or non-symmetric) shape of membrane.

In the case of the symmetric shape of membrane (Eq. (16)), at $F = 0$, there are two possible solutions of Eq. (19):

$$c_{0,1} = \sqrt{\frac{\sqrt{5}}{11}+\frac{37}{132}} \approx 0.6954 \tag{S7}$$

and

$$c_{0,2} = \frac{1}{2}\sqrt{\frac{1}{33}\left(37-12\sqrt{5}\right)} \approx 0.2775 \tag{S8}$$

valid for any d/L. These values correspond to the bending energies $U_{b,\mathrm{s},1(2)} = k_{1(2)}Dw(L-d)/L^2$ with $k_1 = 39.5$ (minimum of $U_{b,\mathrm{s}}$) and $k_2 = 200.5$ (maximum of $U_{b,\mathrm{s}}$). The corresponding geometries are shown in Fig. S4. Importantly, the snap-through transition of membrane takes place as c_0 changes from 0.6954 to 0.2775. We note that Eq. (19) can be used to find F_s needed to 'support' the membrane profile defined by a given value of c_0.

The force as a function of z_{cm} is presented in Fig. S5 for several values of d/L.

Dynamic regime

Here we assume that the membrane transition is induced by an abruptly applied force (similar to the case of our MD simulations). Under the assumption of energy conservation, the threshold switching force can be found from the condition that the initial energy equals the potential energy barrier height (that depends on the applied force).

Technically, the calculation can be performed as follows. The value of parameter c_i, $c_i^*(F)$, corresponding to the maximum of $U = U_b + U_{ext}$ (in the presence of F, see Eq. (18)) can be obtained from $\mathrm{d}U/\mathrm{d}c_i = 0$. Next, $c_i^*(F)$ is substituted into

$$U_b\left(c_i^0\right) + F\zeta\left(c_i^0\right) = U_b\left(c_i^*\right) + F\zeta\left(c_i^*\right), \tag{S9}$$

where c_i^0 corresponds to the potential energy minimum at $F = 0$ (note that $U_b(c_i^0)$ is the energy corresponding to k_1 given below Eq. (S8)). Finally, the threshold switching force is found from Eq. (S9).

If one assumes that, at $t = 0$, the membrane has the non-symmetric profile, then one can get that the dynamic threshold switching force is the same as $F_\mathrm{ns}^{\downarrow}$ given by Eq. (21). This result, however, should only be used as an upper estimate.

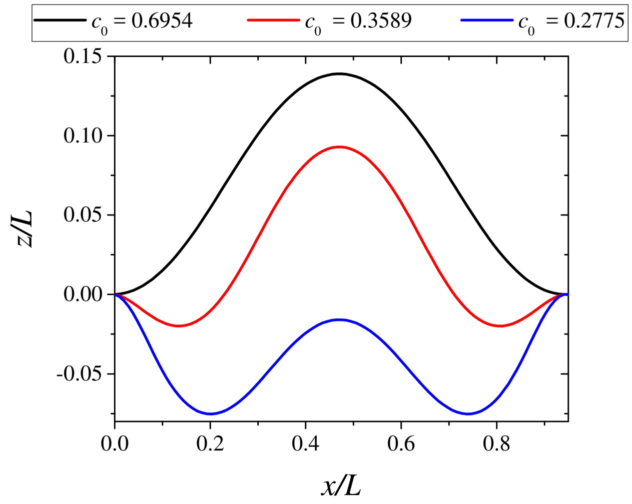

Figure S4. Symmetric shapes of membrane calculated at $d/L = 0.95$. The curves from the top to bottom: the geometry minimizing the bending energy, geometry at the threshold switching force, and geometry maximizing the bending energy.

Indeed, initially, the membrane has the symmetric profile and determining the exact point of switching from the symmetric to non-symmetric shape is a challenge.

Unfortunately, the above mentioned equations can not be solved analytically for the transition through the symmetric profile. Figure S6 shows the numerically found potential energy as a function of position of the center of mass for several representative values of the applied force. This plot demonstrates that the potential barrier height becomes lower than the initial energy when the applied force becomes larger than the threshold switching force F_{sw}.

Various threshold switching forces for the up-to-down transitions are summarized in Fig. 8 together with the results of our MD and DFT calculations. This plot shows that $F^{\downarrow}$ provides the best phenomenological approach estimation for our MD results. The deviation of MD points from $F^{\downarrow}$ curve at smaller d/L can be related to the approximation of small deflections used in our analytical model. Additionally, MD simulations involve energy relaxation channels not included into the simplified model given by Eq. (S9). Overall, however, we are quite satisfied with the results of our phenomenological model.

DFT Calculations

Computational details

Our DFT calculations were carried out using the Jaguar quantum-chemistry[5] package within the electron density functional approach with the use of a Becke three-parameter hybrid functional[6], a Lee–Yung–Parr correlation functional[7] (the B3LYP method), and a 6-31G basis set of atomic orbitals. Optimization of structures was performed by an analytical method up to a gradient of the displacement of atomic positions of 10^{-4} au. The minimum on the potential energy surface was identified by the absence of imaginary values in the matrix of second derivatives. As a test calculation, we have identified that the stretching of an armchair-edged graphene nanoribbon ($L = 20.9$ Å, $w = 14.7$ Å) yields the in-plane stiffness $E_{2D}^{DFT} = 338$ N/m, which is in good agreement with the experimental value $E_{2D} = 340$ N/m.[8]

We investigated a zigzag graphene nanoribbon of the length of $L = 24.6$ Å (10 rings) and of the width of $w = 11.4$ Å. The dangling bonds at nanoribbon boundary were terminated by hydrogen atoms. The hinged boundary conditions were realized by fixing the armchair edges at all calculation steps. The nanoribbon D1 with clamped boundary conditions was constructed from the flattest configuration C1 (with hinged boundary conditions) by an elongation of each armchair side by two rings kept fixed during calculations.

Up-to-down transition

First of all, we note that the optimization of unloaded configurations C1, C2, and C3 gives the bending rigidity $D^{DFT} = 1.75$ eV, which is in good agreement with the experimental value $D = 1.6$ eV[9].

As reported in Ref. [4], while, the non-symmetric path of switching is preferable due to the lower energy barrier, at small deflections from the unloaded state, the nanoribbon tends to be symmetric. A detailed investigation of the up-to-down transition of D1 shows that at small deformations, the difference between the energies of the symmetric and assymetric shapes is small (for instance, the initial displacement of membrane by 0.5 Å requires about 0.02 eV and 0.05 eV for the symmetric and

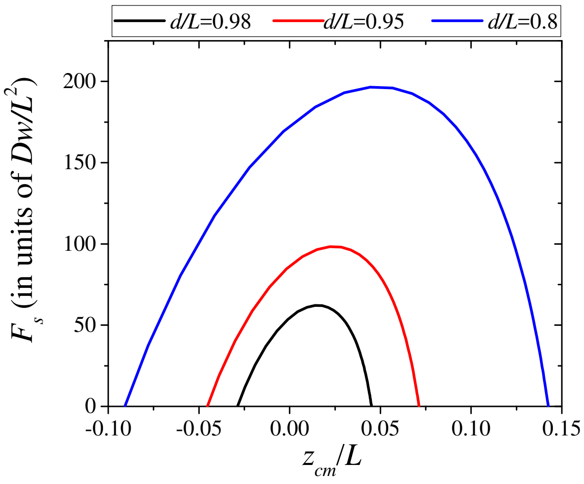

Figure S5. The applied force F_s as a function of z_{cm} found for the symmetric membrane profile in the adiabatic switching regime. The maximum of F_s corresponds to the threshold switching force through the symmetric membrane profile.

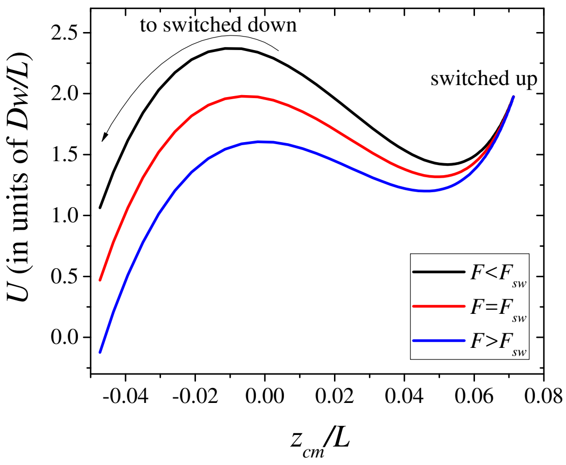

Figure S6. Potential energy landscape for the symmetric switching at several values of the applied force and $d/L = 0.95$.

non-symmetric paths, respectively, see Fig. S7(a)). However, the energy difference between the paths increases significantly with deformation. When the positions of central atoms are close to the level of fixed edges, the deformation energy has a maximum, which was used to estimate the up-to-down threshold switching force[4]. We note that the symmetric path in Fig. S7(a) was generated by an increase of the z-coordinate of central atoms starting at $z \sim 0$.

The deformation energy of buckled graphene membrane with hinged boundary conditions is plotted in Fig. S7(b). In the case of hinged boundary conditions, only the non-symmetric path has been identified in the framework of our DFT approach. It was found that the transition energy barrier is smaller for larger values of d/L.

Overall, the up-to-down threshold switching force estimated from DFT calculations agrees with both MD simulation and elasticity theory results (see Fig. 8). This plot presents the threshold switching force in the units of Dw/L^2 for C1 and C2 configurations. We note that the DFT value for the threshold force for C3 is about $235.3Dw/L^2$. As d/L of C3 is significantly less than 1, the DFT threshold switching force for C3 is not the right quantity to compare with results of the linear elasticity theory.

Stability of Buckled Membrane

The information storage time in the state of buckled membrane is limited by the finite temperature which leads to thermally-activated switching between two stable membrane states at long times. In the classical approach[10], the rate constant k of

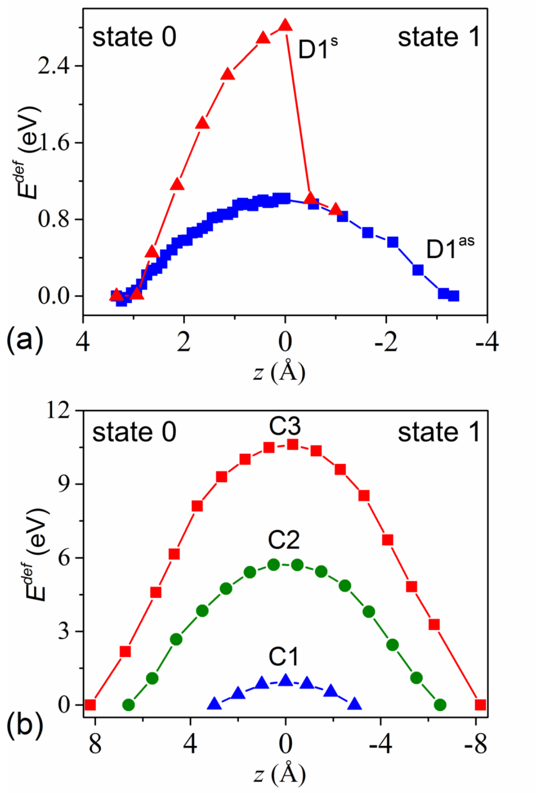

Figure S7. DFT deformation energy versus the position of central atoms in the process of the up-to-down switching. Based on DFT approach, we were able to identify (a) both symmetric and non-symmetric paths for the case of clamped boundary conditions, and (b) only the non-symmetric path for the case of hinged boundary conditions.

thermally-activated switching can be approximately described by Arrhenius equation

$$k = \mathscr{A} \cdot e^{-\frac{E_b}{k_B T}}, \tag{S10}$$

where $\mathscr{A}$ is the pre-exponential factor (according to some more advanced theories[11,12], it describes the energy distribution in the system) and E_b is the potential barrier height. In the case of buckled membrane, E_b corresponds to the energy of the intermediate asymmetric state of membrane during its switching (the saddle point state) with respect to the energy of its stable states.

Next we make an estimation for the thermally-activated switching time of $d/L = 0.94$ membrane of type B (a detailed study of the membrane stability is beyond the scope of this paper). For this purpose we first performed a series of MD simulations of the dynamics of $d/L = 0.98$ membrane without applied force. These simulations have indicated that $d/L = 0.98$ membrane stays in its initial state for at least 10 ns without switching (this is, of course, an underestimate of its storage time). Then, assuming that the change of the pre-exponential factor in Eq. (S10) with d/L compared to the corresponding change in the exponential factor is not significant, and using $E_{0.98} = 0.60$ eV, $E_{0.94} = 1.8$ eV, and $T = 300$ K one finds $k^{-1} > 40,000$ years. Clearly, such stability is sufficient for practical applications.

References

1. Phillips, J. C. *et al.* Scalable molecular dynamics with namd. *J. Comp. Chem.* **26**, 1781–1802 (2005).

2. Vanommeslaeghe, K. *et al.* CHARMM general force field: A force field for drug-like molecules compatible with the CHARMM all-atom additive biological force fields. *J. Comp. Chem.* **31**, 671–690 (2010).

3. Chen, X., Tian, F., Persson, C., Duan, W. & Chen, N.-X. Interlayer interactions in graphites. *Sci. Rep.* **3**, 3046 (2013).

4. Sedelnikova, O. V., Bulusheva, L. G., Okotrub, A. V. & Pershin, Y. V. Spontaneous symmetry breaking during the switching of a buckled graphene membrane. *JETP Lett.* **103**, 244–247 (2016).

5. *Jaguar, version 7.9 (Schrodinger LLC, New York)* (2012).

6. Becke, A. D. Density functional thermochemistry. iii. the role of exact exchange. *The J. Chem. Phys.* **98**, 5648–5652 (1993).

7. Lee, C., Yang, W. & Parr, R. G. Development of the Colle-Salvetti correlation-energy formula into a functional of the electron density. *Phys. Rev. B* **37**, 785–789 (1988).

8. Lee, C., Wei, X., Kysar, J. W. & Hone, J. Measurement of the elastic properties and intrinsic strength of monolayer graphene. *Sci.* **321**, 385–388 (2008).

9. Zhang, D.-B., Akatyeva, E. & Dumitrică, T. Bending ultrathin graphene at the margins of continuum mechanics. *Phys. Rev. Lett.* **106**, 255503 (2011).

10. Atkins, P. & de Paula, J. *Atkins' physical chemistry* (Oxford University Press, New York, 2014).

11. Eyring, H. The Activated Complex in Chemical Reactions. *J. Chem. Phys.* **3**, 107 (1935).

12. Hänggi, P., Talkner, P. & Borkovec, M. Reaction-rate theory: fifty years after Kramers. *Rev. Mod. Phys.* **62**, 251 (1990).
